# The staff perspectives of facilitators and barriers to implementing virtual reality for people living with dementia in long-term care

**DOI:** 10.3389/frdem.2024.1462946

**Published:** 2024-11-15

**Authors:** Joey Wong, Karen Lok Yi Wong, Winnie Kan, Catherine Wu, Mona Upreti, Mary Van, Alisha Temirova, Hadil Alfares, Kayla Wen, Vaishali Sharma, Christine Wallsworth, Jim Mann, Lily Wong, Lillian Hung

**Affiliations:** ^1^School of Nursing, University of British Columbia, Vancouver, BC, Canada; ^2^UBC IDEA Lab, School of Nursing, University of British Columbia, Vancouver, BC, Canada; ^3^School of Social Work, University of British Columbia, Vancouver, BC, Canada; ^4^Faculty of Science, University of British Columbia, Vancouver, BC, Canada; ^5^Faculty of Land and Food Systems, University of British Columbia, Vancouver, BC, Canada; ^6^Faculty of Science, Neuroscience at Simon Fraser University, Vancouver, BC, Canada

**Keywords:** immersive experiences, dementia, Consolidated Framework for Implementation Research, nursing homes, innovation, technology

## Abstract

**Introduction:**

One emerging technology in long-term care (LTC) is virtual reality (VR), an innovative tool that uses head-mounted devices to provide the viewer with an immersive experience. It has been shown that VR has a positive impact on the well-being of residents living with dementia, and staff are essential in the implementation and sustainable use of technology. Currently, there is a lack of inclusion and focus on direct staff perspectives on VR implementation in LTC. This paper aims to report staff perspectives on VR adoption in a 2-year study on a virtual reality program at three Canadian LTC homes.

**Methods:**

Our interdisciplinary team (clinicians, people living with dementia and family partners, trainees, and researchers) explored the facilitators and barriers to implementing VR in LTC, guided by the Consolidated Framework for Implementation Research (CFIR) and intersectionality supplemented CFIR. Twenty-one participants were recruited, including recreation staff, care aides, nurses, screeners, and leadership team members. The team collected data through staff interviews, focus groups, and ethnographic observation field notes. Reflexive thematic analysis was performed to identify themes reporting the facilitators and barriers for VR implementation in LTC from staff perspectives.

**Results:**

The data analysis resulted in three facilitators and four barriers. Facilitators are (1) perceived VR benefits, (2) integrate VR into workflow and routines, and (3) partner with skillful VR champions. Barriers include (1) staff concerns about VR use, (2) financial burden and competing priorities, (3) lack of infrastructure and physical spaces, and (4) staff workload and limited leadership support.

**Discussion:**

This study contributes to the field with staff perspectives on facilitators and barriers to VR implementation. It underscores the rarely discussed aspects of VR implementation, such as funding prioritization and implementation timing. We offer practical strategies to inform future practices and research. Future studies should further explore long-term VR implementation, the involvement of family members as VR facilitators, and the use of VR in LTC.

## 1 Introduction

According to the United Nations in 2020 (United Nations, 2019), the population of older adults is increasing rapidly and is projected to surpass 1.5 billion by 2050. In Canada, the population of people over 65 living with dementia is estimated to be 1.7 million by 2050, with a 65% increase from 2020 (Alzheimer Society Canada, 2022). The older population and individuals living with dementia are heterogeneous regarding different factors such as ethnocultural diversity, indigeneity, and diagnoses (Alzheimer Society Canada, 2024). They have diverse needs that require holistic considerations in care, particularly in LTC homes. Social isolation is prevalent in LTC, especially during the pandemic when there were social restrictions on LTC homes (Boamah et al., 2021; Smith et al., 2022; Wong et al., 2022). Social isolation may adversely impact older adults' mental health, leading to increased rates of depression (Noguchi et al., 2021; Taylor et al., 2018). Thus, technology in LTC has become increasingly prevalent as an innovative solution to enhance the social connections and well-being of residents and older adults living with dementia, such as virtual reality technology (Moyle et al., 2018; Fiocco et al., 2021; Brimelow et al., 2020).

Virtual Reality (VR) technology is increasingly recognized as a valuable tool in dementia research and care. VR is an immersive technology that allows users to experience simulated environments and feel they are a part of the virtual world with various auditory and visual inputs (Appel et al., 2020; Baker et al., 2020). Recent research suggests that VR may offer a promising solution to the issue of social isolation among older adults (Baker et al., 2020; Afifi et al., 2022; Finnegan and Campbell, 2023; Lin et al., 2018; Oppert et al., 2023; Veldmeijer et al., 2020). Other studies indicate VR's positive effects on older adults' physical and mental health, such as physical rehabilitation, preparing appropriate responses to falls through improved balance, enhancing memory and cognition, and encouraging relaxation (Moyle et al., 2018; Chaze et al., 2022; Blomqvist et al., 2021; Chen et al., 2022; Cinalioglu et al., 2023; Molina et al., 2014; Parijat et al., 2015; Seifert and Schlomann, 2021; White and Moussavi, 2016). For older adults with dementia, VR was shown to frequently improve emotional and social well-being by promoting verbal conversation autonomy and decreasing anxiety through enjoyment of virtual environments (Appel et al., 2021a; Mendez et al., 2014). One study reported the enjoyment of VR by participants with severe cognitive impairment (Brimelow et al., 2020).

The integration of VR technology in LTC homes presents unique challenges due to the specific needs of older adults. Studies revealed that older adults face difficulties in using VR, such as operating the technology, fatigue and discomfort while wearing the VR headset and low resolution and limited availability of VR videos (Moyle et al., 2018; Fiocco et al., 2021; Brimelow et al., 2020; Chaze et al., 2022; Rose et al., 2021). In a study of VR with participants living with cognitive impairment in nursing homes (Kim et al., 2021), challenges reported were the need of assistance required to use the VR, the heavy weight of the equipment, difficulties operating VR with limitations in hand movements and limited vision due to participants' reduced visual capacity. One study discussed ethical concerns related to the use of VR by older adults which included how VR could potentially remind them of how they are restricted to the parameters of their care home and might exacerbate social isolation (Brown et al., 2022). Another study assessing ethical considerations on older adults with cognitive impairment using virtual reality-dependent technology elaborated that the use of artificial companions might reduce opportunities for meaningful human interaction (Portacolone et al., 2020). Additionally, a study reflecting on technology ethics and preferences in older adults with and without dementia explained that there are concerns about avoiding infantilization, preserving the autonomy of older adults, as well as a general lack of VR content created specifically for older adults and individuals living with dementia (Diaz-Orueta et al., 2020).

A recent scoping review found that many papers on VR use in LTC focused primarily on residents' experiences (Hung et al., 2023b). To effectively integrate VR in LTC, it is crucial to explore the perspectives of various parties, particularly staff, to better understand the facilitators and barriers relevant to successful implementation. The perceptions of staff are essential, as they are the ones delivering the VR session, especially opinion leaders who informally influence the attitudes and behaviors of their colleagues (Consolidated Framework for Implementation Research, 2024a,b). Additionally, the views of interdisciplinary staff on implementing VR technology in LTC homes are often underexplored. This includes examining how facilitating VR use may influence staff well-being and professional motivation, as well as the role of organizational support, such as leadership support, funding and resources, in VR implementation (Appel et al., 2021b; Orr et al., 2021; Zhao et al., 2021). Moreover, there was more emphasis on challenges faced in implementation rather than the positive experiences and implementation enablers (Kouijzer et al., 2023). Notably, most studies conducted were short-term pilot tests (Chaze et al., 2022; Hayden et al., 2022; Appel et al., 2022). Few reported on VR implementation in LTC over longer periods, nor were the direct experiences of staff using the equipment (Hung et al., 2023b).

### 1.1 The Consolidated Framework for Implementation Research

The Consolidated Framework for Implementation Research (CFIR) (Damschroder et al., 2009, 2022) explains facilitators and barriers to effective implementation. CFIR initially drew from related frameworks and theories from diverse disciplines (Damschroder et al., 2022). This framework is widely adopted in implementation research within healthcare and technology (Means et al., 2020; Schroeder et al., 2022; van Oers et al., 2020). With its comprehensive domains and constructs, CFIR systematically identifies factors influencing implementation across multi-level contexts, from innovation and individuals to outer settings. CFIR was updated in 2022 to include revised domains and constructs (Damschroder et al., 2022). The updated CFIR comprises five domains: The innovation domain looks into the things to be implemented, such as technology, programs, and policies (Consolidated Framework for Implementation Research, 2024c). The inner setting domain examines the implementation setting. The outer setting domain examines the setting outside where the Inner Setting exists—the individuals domain talks about how the roles and characteristics of individuals influence implementation. The implementation process domain is about the implementation activities and strategies. There are a total of 48 constructs across the five domains. Furthermore, there are limited studies exploring the factors impacting VR implementation in LTC and other healthcare settings, especially with the guidance of theoretical frameworks like CFIR (Hung et al., 2023b; Kouijzer et al., 2023). Thus, our study adopted CFIR to systematically enhance our analysis of research findings and identify factors impacting VR implementation in the complex healthcare environment of LTC.

Incorporating an intersectional lens to the CFIR framework can enrich our discussions on VR implementation by considering factors that impact health equity across relevant domains and constructs. Rodrigues et al. reviewed CFIR and identified 28 constructs that could benefit from the incorporation of intersectional considerations (Rodrigues et al., 2023). At the time of the review, the updated CFIR had not yet been published, so some names of domains and constructs differ from the updated CFIR. The resulting intersectionality-supplemented CFIR offers an intersectional perspective, prompting the research team to consider how personal identities and power structures influence the facilitators and barriers to VR implementation (Rodrigues et al., 2023). For example, a resident living with dementia from a cultural minority (the innovation recipients within the individual domain) may find it particularly challenging to access VR as there are only facilitators (the innovation deliverers within the individual domain) who either have knowledge working with people living with dementia or have knowledge working with the cultural group. There is no facilitator who has both areas of knowledge. Yet a facilitator who has both areas of knowledge is needed due to the intersecting identities of the residents living with dementia and coming from a cultural minority. Regarding the power structures, within the inner setting domain in the intersectionality-supplemented CFIR framework, we may ask: *Who holds the power in LTC settings regarding technology implementation?*

Guided by CFIR and the intersectionality-supplemented CFIR, this study provides a systematic interpretation of the facilitators and barriers to implementing VR in LTC homes, considering multi-level influential factors and underlying power dynamics.

### 1.2 Our study

Our qualitative study is part of a larger VR study that explores the implementation of VR technology in three Canadian LTC homes, where over 80 percent of residents have various levels of cognitive impairment. The VR technology that we implemented is a commercialized VR program with a diverse genre of 360-degree videos. The set of VR equipment consists of a facilitating tablet and four headsets. Each LTC home was provided with one to two sets of VR equipment. Guided by the Consolidated Framework for Implementation Research (CFIR) (Consolidated Framework for Implementation Research, 2024c) and the intersectionality supplemented CFIR (Rodrigues et al., 2023), the study aims to answer the research question: What are the facilitators and barriers of VR implementation in LTC from staff perspectives? This study contributes to the literature by expanding the current evidence base regarding VR adoption in LTC. We share practical strategies to inform researchers and LTC leadership teams on future VR and technology implementation to improve the quality of life of people living with dementia in LTC.

## 2 Methods

### 2.1 Research team

Our team comprises one patient partner living with Alzheimer's Disease (JM) and two family partners (CW, LW) with lived experiences with dementia, a researcher (LH) and 10 trainees (JW, MU, KLYW, AT, CW, MV, WK, KW, VS, HA) and three staff champions. We come from diverse backgrounds and hence have varied expertise. Trainees received supervision, guidance and mentorship from LH, our clinical staff champions and our patient and family partners via attending weekly research meetings. They were involved in data collection, analysis, and manuscript writing. Graduate students (JW and KLYW) were involved in coordinating the project and leading manuscript writing under the supervision of LH. Our patient and family partners collaborated with LH in previous projects, so they were experienced in research. They participated in each stage of this study, including research design, data collection, data analysis, and co-authorship of the current paper. Their contribution helped us ensure that the study reflects the needs of people with lived experiences. Additionally, the unique advantage of having patient and family partners work alongside trainees from various disciplines enhanced the robustness of the research by providing diverse perspectives and fostering a comprehensive approach to addressing complex issues.

### 2.2 Study sites, recruitment, and participants

Our study took place in three LTC homes in Vancouver, Canada. Here, we use pseudonyms: Tulip Care Home (*n* = 116), Rose Garden Home (*n* = 132) and Fleetwood Manor (*n* = 156) in the study. The LTC homes have interdisciplinary teams of healthcare providers. The LTC homes were multicultural, with residents and healthcare providers from diverse cultural and language backgrounds. Residents in all LTC homes had complex physical, mental, and/or cognitive comorbidities and hence required 24-h care. About 80% of residents have dementia; 50% of them are in moderate and advanced stages of dementia. We conducted the study in the dementia care units in two homes and the general unit in one of the homes. Staff and volunteers were recruited and trained to facilitate the VR sessions. Each LTC home had a site champion who helped to promote our study to the staff. Research team members met with interested staff to introduce the project and obtain their consent for participation. Following this, the research team member provided in-person trainings on the use of the VR equipment use for staff at the care homes.

Student volunteers were recruited through word of mouth from our research lab members and a university network dedicated to supporting the Parkinson's and Alzheimer's community at the University of British Columbia (UBC). These student volunteers were introduced to the project and signed consent for participation. The research team also provided in-person training on VR equipment to volunteers at the university. Additionally, the volunteers received orientation from clinical staff champions, focusing on how to work with older adults living with dementia in the long-term care homes. The research team visited the LTC homes weekly to support the facilitators for eight months and provided ongoing technical support (January 2022 to August 2022).

### 2.3 Ethics

This study is part of a larger one to understand the implementation and effectiveness of VR in LTC homes. The study received ethics approval from the Ethics Board the UBC Behavioral Research Ethics Board. This paper focuses on the perspectives of staff on the implementation of VR. We obtained verbal and written consent from participants, which included their agreement to publish. To protect participants' identities, all data was de-identified using unique participant pseudonyms.

### 2.4 Data collection and analysis

Purposive sampling was used to recruit staff and volunteer participants who had either facilitated or observed the VR sessions. We continued to collect data until we had sufficient data to answer the research questions. A total of 21 participants were recruited. Research trainees conducted five individual interviews with three recreation staff, a nurse, and a director of care, as well as four focus groups. One focus group involved four volunteers, while the other three comprised of five care aides, a music therapist along with four recreation staff, and two screeners, respectively. The data collection methods (interviews and focus groups) were chosen based on participants' preferences. Both interviews and focus groups were conducted in person or virtually via Zoom meetings. We asked: What were the challenges when implementing the program? How did you resolve these challenges? How could the program be improved? Each interview or focus group lasted about 30 min to 1 h, depending on the participants' availability. Demographics on the staff's disciplines and their role in VR facilitation were collected before the interviews and focus groups. All interviews and focus groups were audio-taped with participants' consent and transcribed. In addition, research trainees went to sites and had regular check-ins with staff. Staff were given a notebook to document their observations. Research trainees also took detailed field notes, documenting their observations during the interviews, focus groups, and check-ins. Our whole research team had weekly meetings about our research for 30 minutes to an hour via Zoom. We discussed the data from interviews and focus groups, as well as stories that might not have been captured in the interviews and focus groups but were observed by healthcare providers and research trainees. We recorded and took notes during these meetings.

We followed an inductive and deductive approach to guide our analysis (Swain, 2018). Our study utilized CFIR and the intersectionality-supplemented CFIR to guide our data analysis. We selected constructs that best aligned with the data through team discussions. Four research trainees (AT, CWu, JW and WK) conducted preliminary data analysis and had five one-hour meetings to analyze the data together from January to April 2024. The four trainees repeatedly listened to the recording and read the transcripts to familiarize themselves with the data, coded the data from the ground, grouped the codes into categories and compared the codes and categories with CFIR to refine the categories. All team members, including patient and family partners, gave inputs to refine the categories and grouped categories into themes, referring to CFIR and the research questions. The team analysis process was iterative, and we moved back and forth between data, codes, categories, themes, and CFIR to reach a consensus on the themes. Our team also discussed the influence of personal identities and power structures on the findings using the intersectionality-supplemented CFIR.

To ensure rigor, our research team constantly encouraged one another to engage in critical reflexivity, examining how our backgrounds, assumptions and social positions influence our actions throughout the research process. During regular team meetings, we posed critical questions and actively discussed and challenged each other's assumptions.

## 3 Results

We interviewed 21 staff, including care aides, a director of care, a music therapist, a nurse, recreation staff, screeners, and volunteers (see Table 1). 14 participants had experience facilitating VR sessions with residents with cognitive impairment or dementia. The other 7 participants had observed residents participating in VR sessions at their workplace. There were also observation and field notes documented by trainees and participants who facilitated the VR sessions. We identified three enablers and four barriers to implementing virtual reality technology in LTC from the staff perspective (see Table 2).

**Table 1:** Demographic table.

**Disciplines**	**Number (*n* = 21)**	**Role in VR facilitation (facilitator/observer)**
Care aides	5	Observers
Director of care	1	Observer
Music therapist	1	Facilitator
Nurse	1	Observer
Recreation staff	7	Facilitators
Screeners	2	Facilitators
Volunteers	4	Facilitators

**Table 2:** Factors influencing the implementation of VR in LTC.

**Facilitators**	**Barriers**
1. Perceived VR benefits	1. Staff concerns about VR use
2. Integrate VR into workflow and routines	2. Financial burden and competing priorities
3. Partner with skillful VR champions	3. Lack of infrastructure and physical spaces
	4. Staff workload and limited leadership support

### 3.1 Facilitators to implementing VR in LTC

Three facilitating factors are identified: (1) perceived VR benefits, (2) integrate VR into workflow and routines, and (3) partner with skillful VR champions.

#### 3.1.1 Perceived VR benefits

The perceived VR benefits are related to two constructs (innovation relative advantage and innovation design) under the innovation domain in CFIR (Damschroder et al., 2022). This theme covers the relative advantages of VR, especially when compared to other activities and technology for residents and how the design interface can facilitate its use in LTC.

##### 3.1.1.1 VR benefits for residents

Staff expressed several benefits of utilizing virtual reality (VR) technology in LTC settings, particularly about residents' physical and mental well-being. Firstly, they highlighted that VR has the potential to motivate physical activity among residents by stimulating head and body movements within the VR environment [Innovation Domain—innovation relative advantage]. This is particularly relevant for residents with dementia, who often experience issues related to physical challenges and minimal communication:

Residents who are immersed in VR exhibit physical responses by moving their hands and heads. This active engagement is a beneficial form of physical activity, as it encourages movements that would not typically occur without such stimuli. (Mandy, Fleetwood Manor, Nurse).

Some staff explained how VR helped calm residents living with dementia by diverting residents' attention from their immediate surroundings to the immersive VR environment. A nurse suggested the potential of VR to reduce the use of antipsychotic medications and support residents' unmet needs [Innovation Domain—innovation relative advantage]:

Residents get frustrated when they can't find their place, and then they get escalated; they feel powerless. So, that causes an increase in medication. By addressing feelings of helplessness, loneliness, and boredom, we can reduce frustration and subsequent escalations that arise from unmet needs. Virtual reality, in my opinion, has the potential to alleviate boredom and, to some extent, feelings of helplessness, particularly considering that many residents desire to explore beyond the care home's confines but are unable to do so. (Alysa, Fleetwood Manor, Director of Care).

Staff also identified the relative advantages of VR implementation compared to conventional activities such as television (TV). Many participants acknowledged that VR offered a unique level of immersion that traditional activities or technology could not provide [Innovation Domain—innovation relative advantage]. Some staff shared that the immersive nature of VR experiences could enhance residents' engagement and attention. One staff said, “Residents haven't had an experience like this before that really feels this real. It [VR] is engaging because you couldn't look away from it, everywhere you looked at was there [in the VR environment].” (Grace, Tulip Care Home, Recreation Therapist).

Staff also appreciated that VR provided an opportunity to include residents with various capacity challenges in novel and immersive activities [Innovation Domain—innovation relative advantage]. A recreation staff shared an example of a resident who was deaf, communicating solely through sign language and usually isolated in her room. When engaging in one-on-one VR experiences, the resident became joyful, expressive, and engaged through sign language. She even created a unique “sign” for VR whenever she wanted to participate.

##### 3.1.1.2 VR benefits for staff

Staff added that the implementation of VR technology in LTC homes had positive implications for the staff's work environment and job satisfaction. They observed that VR served as a tool to facilitate more meaningful interactions with residents and offered a refreshing break from routine tasks, contributing to a more dynamic and enjoyable work experience [Innovation Domain—innovation relative advantage]. A recreational staff, Margaret, expressed, “It's very different from what we do daily. I think it's a nice break for them to try something new. For the residents and for staff too.” She further added, “I enjoy the reactions. She [a resident] was in a wheelchair, but she's moving her chair around, reaching out, talking in her language to the VR video… watching her reaction brings smiles to staff and residents.” (Margaret, Fleetwood Manor, Recreation Staff).

Furthermore, participants mentioned specific instances where VR technology could enhance their interactions with residents [Innovation Domain—innovation relative advantage]. A music therapist incorporated VR to enhance the therapy process in a dementia unit by playing Chinese music when a resident watched a video about Hong Kong. The therapist also observed the same group of residents consistently participated in the therapy activity, with an increase in the number of residents joining the session each time. A volunteer saw VR as an additional activity to volunteer visits in the care home:

The conversations [between volunteers and residents] are also limited to things like the weather. The residents can't relate to much like the news outside, and volunteers don't know much about them. But I feel like with the VR session, you can have many more conversations and get to know the residents on a much deeper level. (Jessica, Rose Garden Home, Volunteer).

Staff outlined the ease of VR setup as a crucial factor influencing their attitudes toward adopting VR [Innovation Domain—innovation design]. A recreation staff commented, “It was very easy for me to understand the software and implement it with the resident.” (Grace, Tulip Care Home, Recreation Therapist). Regarding the design and interface of the VR equipment, she added, “I find the VR equipment easy and straightforward to use. The layout of everything, the menu on the tablet, and the way it's set up are just like a Netflix menu you just scroll through.” (Grace, Tulip Care Home, Recreation Therapist).

#### 3.1.2 Integrate VR into workflow and routines

The staff mentioned two other factors that could positively impact VR adoption in LTC: modifying the VR session schedules and creating a plan for usage. These factors are connected to the constructs (adapting and planning) under the implementation process domain in CFIR (Damschroder et al., 2022).

The impact of routine VR sessions was significant when VR sessions were incorporated into the weekly recreational activity schedules at the LTC sites [Implementation Process Domain—adapting]. This helped build familiarity through repeated, consistent exposure to VR equipment and facilitators for the VR sessions. Staff working in the dementia unit observed residents' anticipation of the VR sessions: A resident immediately approached me and sat down and put on a headset by himself while I was setting up for a session (Observation note, Fleetwood Manor). The staff wrote another observation: A resident called me over to put on the headset [when I arrived at the unit with the headsets] (Observation note, Fleetwood Manor). Consequently, there was often a steady group of engaged residents in the VR sessions. Staff expressed that they were able to learn more about the preferences and dislikes of each resident, thus being able to tailor the experience to a higher degree.

The timing of the VR usage and sessions was another factor that would facilitate the usage of VR [Implementation Process Domain—adapting and planning]. A nurse mentioned that matching the residents' routines to VR usage would be helpful to support VR adoption. For example, weekends were days when residents needed recreational activities while families would visit to join. Besides aligning with LTC routines, a recreation staff articulated the alignment of VR activity with the seasonal changes and weather: VR can be used more frequently in winter and rainy days when residents can't go outside. (Floria, Rose Garden Home, Recreation Staff).

#### 3.1.3 Partner with skillful VR champions

Staff participants articulated from the examples they shared the importance of having a suitable facilitator who knew the residents well, shared the same cultural background and spoke the same languages as the residents. Facilitation competency and knowledge of the use of VR equipment were two significant characteristics of skillful VR facilitators. This theme about VR facilitators is tied to the innovation deliverers construct under the individuals domain of CFIR. This theme covers the characteristics and capability of the VR facilitators who directly deliver the innovation (Damschroder et al., 2022).

Most staff agreed that facilitators would feel more comfortable supporting the VR session when they knew the residents' backgrounds [Individuals Domain—innovation deliverers]. A music therapist shared that he could use VR to prevent the escalation of residents living with dementia. Another therapist would play Chinese music with a video about Hong Kong on a VR headset to comfort a Chinese resident. A care aide explained how familiarity with certain residents helped with her use of VR. She said, “I used the VR with the residents in the dementia units because I work on these floors a lot. I know those residents. I know who likes what, so I can easily facilitate the VR session.” (Bonnie, Fleetwood Manor, Care Aide).

Besides the knowledge of the residents' backgrounds, having a shared cultural and language background is considered instrumental in a better VR experience for residents. Some staff members shared that not speaking the same language was a barrier to social connection during VR sessions. A recreation staff shared that family members could be a good resource as they could help translate the conversations in the VR session, which encouraged socialization [Individuals Domain—innovation deliverers]. A staff member's observation echoes this sharing: The residents would be more comfortable trying the VR equipment if the family members facilitated the session, and they would interact differently, compared to facilitators who did not speak their language (Field note, Tulip Care Home). A recreation staff shared an observation when the Hindi-speaking facilitator spoke in Hindi, the resident was happy and enjoyed talking about her fond memories of her language, e.g., family trips and visiting her hometown.

**Figure 1:**
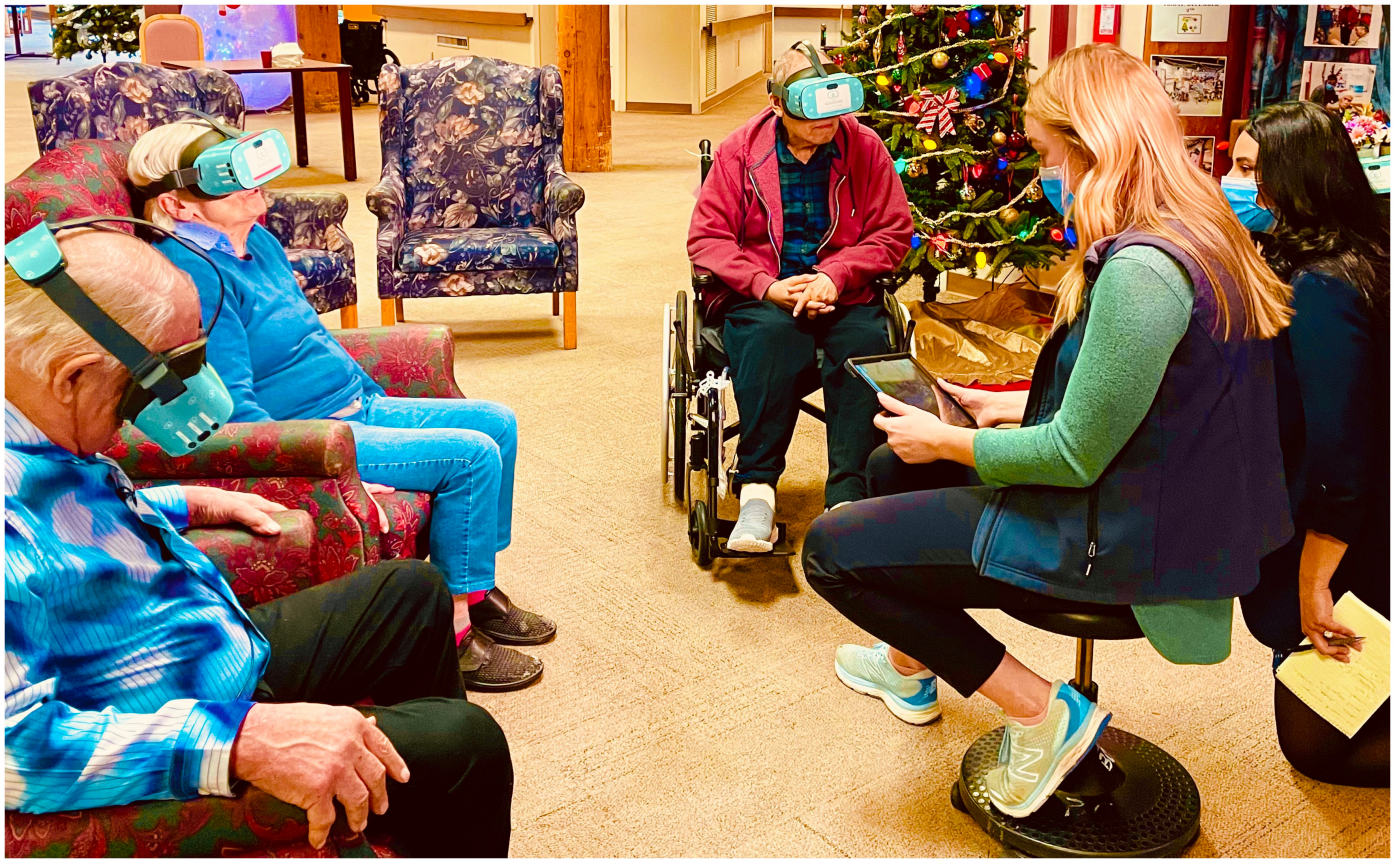
A staff facilitating a group VR session.

Some staff also mentioned the competency to facilitate and use the equipment will enhance residents' experiences in the VR session. The facilitator's skills to encourage interactions were noted as vital [Individuals Domain—innovation deliverers]. A staff shared, “Some staff find it harder to manage a group vs. one-on-one. It's hard to find people to have the skills to facilitate groups.” (Raymond, Rose Garden Home, Recreation Staff). Figure 1 shows a staff facilitating a group VR session.

### 3.2 Barriers to VR implementation in LTC

Despite the advantages, staff shared barriers that might hinder VR usage, sustainability, and adoption in LTC. Four impeding factors are identified: (1) staff concerns about VR use, (2) financial burden and competing priorities, (3) lack of infrastructure and physical spaces, and (4) staff workload and limited leadership support.

#### 3.2.1 Staff concerns about VR use

Staff concerns cover the constructs innovation design and innovation complexity under the innovation domain that explain potential barriers to implementing VR in LTC (Damschroder et al., 2022).

One concern raised by staff around VR use was related to headset discomfort [Innovation Domain—innovation design]. The staff mentioned that some residents experienced discomfort wearing VR headsets. A recreation staff noted, “People would be sweating, and they'd have the marks dug into their face. But then if the VR headset is not on tight enough, it's out of focus” (Grace, Tulip Care Home, Recreation Therapist). Another staff shared, “I wish the [VR] headset was lighter and more comfortable for residents, especially for those with glasses.” (Nancy, Rose Garden Home, Recreation Staff).

Additionally, participants expressed that the VR program implemented with visual and audio stimulations may not be the only best option when compared to activities that are more tactile and provide other sensory stimulations [Innovation Domain—innovation design]. A staff shared, “Compared to things like bowling, VR just isn't physical nor touchable.” (Scott, Tulip Care Home, Recreation Therapist).

Staff commented on the time needed to manage the video library for the VR equipment [Innovation Domain—innovation complexity]. A staff said, “Downloading videos to the tablet from the database, looking through the database and choosing the ones you want are time-consuming” (Grace, Tulip Care Home, Recreation Therapist). In addition, staff worried about the cleaning procedures associated with VR equipment. A recreation staff expressed apprehension about the complexity of cleaning VR headsets and ensuring proper hygiene practices [Innovation Domain—innovation complexity].

#### 3.2.2 Financial burden and competing priorities

This theme covers barriers related to the innovation cost under the innovation domain, funding, and relative priority under the inner setting domain regarding the available resources and sustainability of VR equipment use in LTC (Damschroder et al., 2022).

Staff expressed concerns about the cost of the VR equipment and the cost of integrating VR into the care homes [Innovation Domain—innovation cost]. The perceived cost of the VR headsets deter care staff from facilitating VR sessions. Many are worried about damages and liability. One care aide expressed, “The VR equipment is expensive, so we have to be careful with them” (Irene, Fleetwood Manor, Care Aide), while another care aide added, “I don't want to hurt the machines.” (Lucy, Fleetwood Manor, Care Aide).

There is also pervasive uncertainty regarding the sustainability of long-term VR adoption, stemming predominantly from the perceived financial burden, encompassing both initial acquisition and ongoing operational expenses [Inner Setting Domain—Funding]. The substantial investment required to sustain VR adoption rendered introducing this technology challenging in the care home community. A Director of Care shared,

I wasn't sure if I should introduce this VR program because we didn't have the funding, and I didn't know how long this would be in our care home. So, it is also harder to bring the idea to the board of directors. It's too expensive with a subscription cost. (Alysa, Fleetwood Manor, Director of Care).

She further articulated considerations over the cost of VR technology by emphasizing the competing priorities faced by care homes [Inner Setting Domain—relative priority]. She highlighted the scarcity of available funding and the necessity to allocate resources judiciously across various activities and expenditures.

#### 3.2.3 Lack of infrastructure and physical spaces

The information technology infrastructure and physical infrastructure under the inner setting domain are relevant to this theme of how these infrastructures in LTC impede VR implementation (Damschroder et al., 2022).

Across all three LTC homes, the lack of a stable Wi-Fi connection emerged as a significant barrier, causing frustration among staff members who were required to connect each headset to Wi-Fi before every session manually [Inner Setting Domain—information technology infrastructure]. Furthermore, the frequent internet disconnections during sessions were disruptive and further complicated the challenge [Inner Setting Domain—information technology infrastructure]. Consequently, some staff resorted to using their personal mobile data to facilitate sessions or, in some cases, felt discouraged from utilizing the technology altogether.

Physical space limitations within the care homes posed another obstacle, hindering the VR equipment's utilization to its optimal potential [Inner Setting Domain—physical infrastructure]. As a recreation therapist remarked:

It was hard for us to find a safe and accessible space where the care aides could grab the VR equipment because we had to lock it in a nursing cupboard. We can't leave expensive equipment lying around and it became a barrier to the care aides to access and implement it. (Grace, Tulip Care Home, Recreation Therapist).

Additionally, the constrained physical spaces within the care homes presented challenges for conducting larger group sessions [Inner Setting Domain—physical infrastructure]. A volunteer expressed, “If you wanted to do a group session, [the spaces] are pretty cramped or have a lot of tables and objects, it's kind of hard to get the residents together.” (Judy, Rose Garden Home, Volunteer).

#### 3.2.4 Staff workload and limited leadership support

This theme including staff workload and leadership support belongs to the work infrastructure and culture constructs under the inner setting domain regarding staffing levels and organization of tasks among individuals and disciplines (Damschroder et al., 2022).

One challenge encountered during VR implementation was the heavy workload of care home staff [Inner Setting Domain—work infrastructure]. According to recreation team members, integrating VR sessions into their activity schedule was manageable. However, care staff such as care aides and nurses faced notable hurdles due to time constraints, conflicts in care routines and overwhelming care duties before considering VR with the residents.

Care aides, who were initially keen to be engaged in VR implementation, feared being perceived as neglecting their duties or burdening colleagues with additional tasks if they shifted their attention to VR sessions with residents [Inner Setting Domain—work infrastructure]. Many declined project involvement while awaiting their managers' guidance before committing. This sentiment was echoed by recreation staff, who noted the absence of management support or guidance for care staff to engage with VR: “I don't think there was a lot of support from our management to help them use it. They weren't creating extra [dedicated] time for the care staff to be able to use it.” (Grace, Tulip Care Home, Recreation Therapist).

Along with the challenges of creating changes in busy workspaces in LTC homes, fostering a culture of readiness for the change emerged as a foundational factor in encouraging staff to embrace VR technology and implement it into their workflow [Inner Setting Domain—culture]. As articulated by a Director of Care:

The problem with when you want to create change is that, first of all, it's hard to make changes. People need to have a buy-in, which can be difficult […] I think that when you want to bring virtual reality, that's a new thing that you're adding into the loop. So, you need to have education and change the mindset of people, processes, and everything else. (Alysa, Fleetwood Manor, Director of Care).

## 4 Discussion

Our results described the facilitators and barriers to implementing VR technology from staff perspectives in three Canadian long-term care, specifically for residents living with cognitive impairment and dementia. In the discussion, we will discuss the facilitators, barriers and potential strategies guided by four domains (innovation, inner setting, individuals, and implementation process domains) in the CFIR framework. We will also explore the potential influence of personal identities and power relations in LTC with the guidance of the intersectionality-supplemented CFIR.

### 4.1 Innovation domain

Under the innovation domain, the advantages, design, and cost of VR technology were discussed from the staff's perspectives.

Our results showed that staff acknowledge the positive impact of VR technology on residents, particularly residents with dementia who might not always engage in recreation activities in the care homes. For example, some staff mentioned that VR technology could encourage residents to move their head and neck, alleviate residents' boredom and act as a distraction for some residents experiencing behavioral challenges. The immersiveness of VR was perceived as its relative advantage. A study by Freiesleben et al. (2021) shared that unclear benefits of technologies could be a barrier to adopting technologies. The acknowledgment of VR's unique benefits can be spread to motivate other staff to use this technology with other residents. While some staff were excited and considered VR as something new and novel to the care homes, the novelty effect of this innovative technology may diminish over time. It is crucial to incorporate appropriate strategies to sustain the use of technology (Jeno et al., 2019; Shin et al., 2018; Miguel-Alonso et al., 2024). For example, VR implementors such as healthcare leaders can continuously engage staff to understand their needs and share stories of how VR positively impacts the residents' quality of life and staff's job satisfaction.

Similar to studies introducing innovative technologies such as telepresence robots to LTC homes, some staff felt nervous and worried when working with these new technologies (Ren et al., 2024). This nervousness and unfamiliarity often lead to hesitancy and reluctance to adopt technology if no appropriate support is given. Having easy-to-use technology devices and proper structural support could enhance the technology implementation (Ren et al., 2024). Our study highlighted staff concerns with the everyday maintenance and infection control of VR equipment. Sharing clear information on maintenance and care of the equipment can potentially impact staff buy-in and adoption of VR in LTC.

Staff concerns about VR design, such as the potential discomfort of wearing the headsets, echoed the findings from another VR study (Kim et al., 2021). Frontline staff also expressed their concern about the equipment cost and the impact of the cost on VR use in our study. Interestingly, the cost of VR equipment and programs is rarely discussed as a concern in the literature on VR use in LTC. In contrast, the literature focused on how technological advancement has reduced the cost of VR technology in the marketplace (Langlet et al., 2021; Bryant et al., 2020). Our study showed that the perceived highly valued equipment is still a concern for staff and may create an accountability burden for staff. The perceptions of the staff might lead to a possible exclusion of some residents living with dementia whom the staff might suspect are at risk of damaging the equipment. This, in turn, excluded these residents' potential to benefit through engaging with VR technologies. The leadership teams need to support staff in using the equipment comfortably. They can co-design strategies with staff to enhance the VR programs' inclusiveness for residents with different stages of dementia and diverse needs.

Notably, staff compared VR with traditional forms of resident engagement, such as bowling and other tactile activities that provide other sensory stimulation. This underscores that VR should not be promoted as a replacement, but rather as a supplement to enhance the quality of life for residents living with dementia, alongside the existing individual and group indoor activities and outdoor visits. Additionally, there is potential for more creativity in using VR in LTC homes. For example, staff could co-plan VR activities that incorporate additional sensory elements, such as smell and touch.

### 4.2 Inner setting domain

Some important considerations under the inner setting domain were around the funding prioritization and marketing price of VR technology, equitable residents' access to VR technologies, and work infrastructure in LTC.

Staff and leadership team participants in our study expressed financial concerns about using VR technology in LTC due to the limited organizational funding and different prioritized initiatives. There were challenges to sustaining VR use financially despite potential benefits to residents. The leadership's hesitancy to adopt VR due to concerns about the equipment and maintenance expense echoes some literature on VR technology adoption in other healthcare settings, e.g., mental health (Chung et al., 2022) and rehabilitation settings (Bryant et al., 2020). The funding prioritization in LTC usually relies on the decisions of leadership teams and the board of directors. By applying an intersectional lens, this prioritization process reveals the power relations and potential power imbalances in technology implementation in LTC (Rodrigues et al., 2023). The values and perceptions of decision-makers regarding residents' capacity to use technology such as VR will significantly impact the implementation and adoption of these tools. There is an urgent need to ensure equity in LTC by engaging residents and relevant parties in these important meetings to make decisions about residents' needs and meaningful activities in LTC. Healthcare leaders must acknowledge whose voices are at the decision table and whose voices are missing with an intersectional lens and reflect on the norms and power structures in LTC homes where residents' voices may not always be present in the planning and funding prioritization decisions (Rodrigues et al., 2023). An example of upholding inclusivity is shown in a recent study in which the citizen panelists shared key insights on the effectiveness of having advisory boards involving residents and families to inform and support decisions in LTC (Wilson et al., 2022).

Our results showed that the funding prioritization in LTC was closely tied to the marketing price of technology. This brings attention to the need to explore the innovation ecosystem in healthcare. The collaborations between industrial parties, researchers and LTC healthcare settings should not be overlooked. These “public-private partnerships” and “university-healthcare settings-industry collaboration” can potentially help adapt and build business models that work for the healthcare system (Abeykoon, 2021) and support lower cost and risk sharing (Huynh, 2024). This collaboration can help strive for a “balance between social and financial outcomes” (Angeli and Jaiswal, 2016, p. 489). Industrial parties could arrive at a middle ground when setting the marketing price, thereby enhancing the spread and sustainable use of the developed innovation and maximizing its product's potential to improve technology access and residents' quality of life in LTC.

The information technology and physical infrastructures in the LTC setting can also impact residents' equitable access to technologies. Similar to literature related to technology implementation (Ren et al., 2024; Hung et al., 2022; Hoel et al., 2022), staff shared that an unstable Internet connection could hinder the implementation of VR technology in LTC. Some staff mentioned how VR technology could be a great add-on to the limited activities in LTC that were meaningful for people with dementia. Having a stable Internet connection will ensure opportunities for residents living with dementia to access these technological activities. The leadership teams can evaluate the organizational readiness for technology implementation by testing the Internet connection speed and addressing potential barriers to using the technology prior to the technology implementation.

Physical space is another factor to be considered regarding VR implementation. Miller et al. (2024) mentioned the importance of having a safe area for VR use with residents in aged care settings. Our study participants shared further insights on the available spaces for storing the VR equipment, which is less frequently mentioned in the literature. Although VR headsets and the equipment do not occupy much space, some staff shared challenges in finding a safe and easily accessible space for storing the equipment and access by multidisciplinary staff. With the increasing use and advancements of technologies in LTC homes, there need to be discussions, planning, and considerations for a designated space to store and recharge technology equipment that is safe and accessible for interdisciplinary staff.

Our staff participants often mentioned the need for staffing and assignment of roles for VR adoption. Besides its use in recreational activities, VR technology also has a potential therapeutic impact on residents living with dementia, which can be incorporated into the care routines by nurses and care aides. However, from the literature findings and our participants' sharing, technology implementation in LTC is challenging due to an understaffed environment with staff burnout in LTC (Ren et al., 2024; Wilson et al., 2022). In our study, although staff were interested in using VR headsets with residents and knew the benefits of VR, they needed management support in VR implementation. Having protected time allows staff to feel recognized and valued for using the VR technology with residents and avoids potential conflicts among staff regarding workload. This aligns with an article by Miller et al. (2024) that described dedicated time for designated staff as a necessary component to implement a VR-based activity program in aged care settings. Staff concerns about potential conflicts with colleagues highlight the underlying power dynamics among staff and their agency in using technology with residents, which will influence the implementation of VR. Additionally, there is a need for leadership teams, staff, and researchers to reflect on and recognize the intersection of power dynamics and the working cultural context of diverse disciplines. This awareness is essential to fostering effective communication and supporting technology use in LTC (Rodrigues et al., 2023).

### 4.3 Individuals domain

The person facilitating the VR session, i.e., the innovation deliverers, plays a significant role in implementing VR in LTC. A staff participant in our study shared the importance of the VR facilitators' skills and knowledge about the residents in supporting residents in using VR technology in LTC. How LTC homes support VR implementation by involving skilled and culturally appropriate facilitators reflects the extent to which these organizations prioritize the diverse perspectives and needs of residents (Rodrigues et al., 2023).

In a recent study by Brimelow et al. (2020), the leisure and lifestyle coordinator, registered nurses, and personal carers were the VR facilitators and supported residents in choosing their preferred VR programs. The group dynamics created by the facilitators in VR sessions enhanced residents' enjoyment of the VR sessions (Brimelow et al., 2020). With the knowledge about the residents, facilitators could show them relevant VR videos and support residents in conversations such as talking about their past. Coelho et al. (2020) shared that participants living with dementia used VR headsets to visit meaningful locations and reminisce about past events. VR reminiscence therapy can enhance the cognitive and psychological well-being of residents living in LTC (Khirallah Abd El Fatah et al., 2024).

Staff also mentioned the impact of having a VR session facilitated by individuals who share similar cultural backgrounds and speak the same language as the residents, in order to meet the needs of the heterogenous population in LTC. Within the individuals domain, residents are considered the innovation recipients and VR facilitators are innovation deliverers. Both of their unique personal identities and the intersection of these identities should be considered in the implementation of these innovations. For example, VR facilitators from the same cultural background would understand the place the resident described during the VR session. Facilitators speaking the same language and having knowledge of dementia can help overcome the communication barriers to support the socialization of residents living with dementia in the VR session. This is echoed by a review by Martin et al. (2018), which shared the impact of culturally and linguistically congruent care on LTC residents' well-being. The intersectionality perspective emphasizes the intersection of personal identities. Facilitators should also consider identities beyond residents' cultural backgrounds and languages, including their social positions, varying stages and types of dementia, and gender. These factors can significantly influence their perceptions of new technology and their experiences with specific genres of VR videos. By acknowledging these identities, facilitators can help enhance residents' VR engagement. Additionally, facilitators should reflect on how their own positions, experiences, and intersecting identities shape their assumptions about the residents' capacities and interactions with VR. These assumptions can create barriers to successful VR implementation.

Speaking the same language enhances understanding among residents living with dementia, making family members potential facilitators in VR sessions. In a study by Luijkx et al. (2015), family members, including spouses and grandchildren, have played a significant role in implementing technology for community-dwelling older adults. Older adults were more willing to accept the technology, and spouses could support each other in using the technology. However, previous studies did not describe the process and impact of the involvement and facilitation in VR implementation by family or facilitators with the same cultural and language background in LTC. There is potential for researchers to leverage family members' strengths in facilitating the VR program in LTC. For example, family members from different cultures can be trained as VR facilitators to support the use of VR and orientate other family members interested in facilitating VR use in LTC.

### 4.4 Implementation process domain

Our results underscore the importance of adapting the VR program to the work routines in LTC and residents living with dementia.

Seldomly mentioned in other literature, our staff participants suggested “timing” as an essential consideration in the uptake of VR technology. One staff mentioned that VR technology was beneficial during wintertime when residents could not visit the garden or have outdoor visits. VR technology can potentially supplement the existing program in LTC when outdoor activities are not appropriate due to weather and outdoor spaces, e.g., cities having a longer period of extreme cold or a lack of outdoor spaces. Furthermore, it is essential to understand staff and residents' routines while planning the VR sessions to achieve the optimal use of the technology in LTC homes. Conflicts between residents' daily routines were a challenge noted in technology implementation in a recent study in LTC (Hung et al., 2023a).

For residents living with dementia, it is also essential to have a routine VR implementation to build their familiarity with the VR technology and the VR facilitators. Routine activities allow residents living with dementia to better adapt to technologies. This echoes an example in the literature where a family member called in every day through a telepresence robot to a resident living with dementia. The calls became the resident's routine with improved engagement with the technology over time (Hung et al., 2023a).

Table 3 is a summary of the facilitators, barriers, and potential strategies for VR Implementation under relevant CFIR domains and constructs.

**Table 3:** Summary of facilitators, barriers and potential strategies for VR implementation.

**Facilitators**	**CFIR domain**	**Construct**	**Potential strategies for VR implementation**
Perceived VR benefits	Innovation domain	Innovation design	• Share the unique benefits of VR technology to residents living with dementia in LTC through different communication channels to staff • Encourage staff to observe or facilitate VR sessions with residents to have first-hand experiences of the impact on residents • Encourage inclusion and equity of VR technology use for residents with different stages of dementia
		Innovation relative advantages	
Integrate VR into workflow and routines	Implementation process domain	Planning	• Plan the time for implementation includes seasonal considerations, staff working routines and residents' activity routines • Acknowledge that the VR technology is a supplementary activity • Having routines in the VR programs can enhance residents' adaptations to the technology
		Adapting	
Partner with skillful VR champions	Individuals domain	Innovation deliverers	• Ensure adequate training for VR facilitators in LTC to enhance competency with the technology and facilitation skills in the VR sessions • Explore the inclusion of VR facilitators with similar cultural backgrounds with residents, e.g., family members • Support family members to orientate other family members for VR adoption
**Barriers**	**CFIR domain**	**Construct**	**Potential strategies for VR implementation**
Staff concerns about VR use	Innovation domain	Innovation complexity	• Consider the complexity of technology and plan supportive strategies in logistics and maintenance of VR technology
		Innovation design	• Engage staff to co-design strategies to enhance the VR program to be more inclusive for residents and to increase staff comfort in using the equipment with residents with diverse needs
Financial burden and competing priorities	Innovation domain	Innovation cost	• Be aware of whose voices are on the decision table and whose voices are missing when it comes to funding decisions and prioritizations in LTC settings • Encourage collaboration between industrial parties, researchers and LTC healthcare leaders to achieve social and financial outcomes
	Inner setting domain	Funding	
		Relative priority	
Lack of infrastructure and physical spaces	Inner setting domain	Information technology infrastructure	• Advocate for the basic needs of stable Internet connection in LTC homes • Ensure designated safe and accessible space for technology storage and charging • Evaluate organizational readiness in implementing technology, e.g., testing the Internet connection speed
		Physical infrastructure	
Staff workload and limited leadership support	Inner setting domain	Work infrastructure	• Adequate management support across disciplinary teams for VR technology implementation, e.g., initiate protected time for staff to facilitate VR sessions, discuss with interdisciplinary teams to include VR facilitation in the work schedules • Ensure clear communication on the support for VR technology use in LTC homes • Acknowledge diverse working cultures of different disciplines
		Culture	

### 4.5 Implications for future research

Based on the findings, researchers should consider utilizing frameworks such as CFIR and the intersectionality-supplemented CFIR framework throughout the research process, starting from the planning and design stages of future studies on VR use in LTC. To address the cultural backgrounds of the residents, facilitators who share the same language and cultural backgrounds, such as family members, should be recruited to facilitate the VR sessions. Involving multidisciplinary staff and leadership team in planning and co-designing the VR implementation, while recognizing the power relations in the team, will support the VR adoption in the care home. Moreover, understanding the underlying assumptions of staff and the organization can help improve equitable access to technology for residents living with dementia. Researchers should prioritize the voices of residents living with dementia and staff, who are often undervalued and underrepresented in LTC research.

For future studies, researchers can document and share the process of involving staff from diverse disciplines and family members as facilitators in VR sessions. Studies can examine the cost and decision-making process of using VR technology in LTC. There can be case studies to share the impact, challenges and process of engaging residents living with dementia in the funding allocation and prioritization for implementing technologies in LTC settings. Studies can also compare the differences in the role and uptake of VR technology in urban areas and areas where outdoor activities are unfavorable for residents. A longitudinal study can be done to explore the sustainability of VR programs in LTC settings.

### 4.6 Strengths and limitations

The unique strength of the study is its contribution to the field with the unique insights and direct voices from LTC multidisciplinary staff, which are generally understudied and poorly understood. The team also engages people living with dementia and family partners in the research process, including the team data analysis. Our study implemented VR technology for eight months to explore the staff's experiences with VR implementation in LTC homes. The evidence-based framework - CFIR with an intersectional lens guided our data analysis and discussions. Our study has three limitations: Due to the staff shortages in the care settings, there were limited opportunities for care staff (nurses and care aides) and disciplines such as physiotherapists and occupational therapists to deliver the VR sessions. Although our research team tried to engage these disciplines, they were unable to participate in our research due to workload and busy schedules. There would be richer content if the care aides and nurses could participate more in facilitating the VR sessions. The experiences and insights from some participants were based on their observations instead of direct VR facilitation. Moreover, our research team only collected demographics regarding the participants' disciplines and their role in VR facilitation, which limits the analysis of other characteristics, such as cultural backgrounds and gender, that may affect staff perspectives of VR implementation in long-term care. Furthermore, the study results were based on three Canadian LTC homes in urban areas, where the experiences in LTC homes in rural and other areas have yet to be explored due to the potential differences in preference and needs on VR videos of residents living in those areas.

## 5 Conclusion

VR technology can potentially improve the quality of life of residents living with dementia in LTC, and staff perspectives are crucial to successful technology implementation. The study discusses the facilitators and barriers to implementing VR technology in LTC homes from the perspectives of interdisciplinary staff from three Canadian LTC homes with the guidance of the CFIR. The results highlight the seldomly mentioned aspects such as the impact of costs, funding prioritization and the timing of VR implementation. Future researchers and leadership teams can consider the practical strategies offered in our study to guide the implementation of VR technology to improve the quality of life of residents with dementia in LTC homes.

## Data Availability

The raw data supporting the conclusions of this article will be made available by the authors, without undue reservation.
